# Impact of the use of illicit and licit substances and anxiety disorders on the academic performance of medical students: a pilot study

**DOI:** 10.1186/s12909-022-03752-6

**Published:** 2022-09-19

**Authors:** Pedro Marques Ferreira, Rauni Jandé Roama Alves, Denise Engelbrecht Zantut-Wittmann

**Affiliations:** 1 Medicine Course, Faculty of Health Sciences, Federal University of Rondonópolis, Rondonópolis, Brazil; 2 Psychology Course, Institute of Human and Natural Sciences, Federal University of Rondonópolis, Rondonópolis, Brazil; 3 Division of Endocrinology, Department of Internal Medicine, Faculty of Medical Sciences, State University of Campinas, CampinasCampinas, Brazil

**Keywords:** Academic performance, Anxiety disorders, Substance use

## Abstract

**Background:**

Medical students have a considerable prevalence of anxiety and substance use disorders. Our aim was to assess the presence of anxiety disorders and the use of alcohol and licit and illicit substances, and their influence on the academic performance of medical students.

**Methods:**

This is a cross-sectional, non-experimental pilot study, with quantitative analyses, in which 67 medical students at the Federal University of Rondonópolis, Mato Grosso, Brazil (UFR), were evaluated through the application of non-invasive anxiety assessment instruments, screening for involvement with tobacco, alcohol and other substances, related to school performance coefficient, between June 2019 and March 2020.

**Results:**

Worse academic performance was associated with frequent use of tobacco and its derivatives (*p* = 0.0022), marijuana (*p* = 0.0020), hypnotics and sedatives (*p* = 0.0138). Also, the performance was negatively correlated with the use of tobacco (*p* = 0.0004), alcoholic beverages (*p* = 0.0261), cannabis (*p* = 0.0075), sedatives (*p* = 0.0116) and trait anxiety (*p* = 0.0036). Greater trait anxiety intensity was associated with previous use of tobacco (*p* = 0.0276), marijuana (*p* = 0.0466), amphetamines/ecstasy (*p* = 0.0151), and hypnotics/sedatives (*p* = 0.0103). State anxiety was positively correlated with heavy alcohol use (*p* = 0.0434). Higher state anxiety intensity was related to needing intervention due to the use of amphetamines/ecstasy (*p* = 0.00379). Students from the intermediate classes of the course (3rd and 4th years) had a higher frequency and intensity of use of tobacco and its derivatives (*p* = 0.0133), amphetamines or ecstasy (*p* = 0.0006), and inhalants (*p* = 0.0256).

**Conclusions:**

Worse academic performance in medical students was correlated with licit and illicit substances use and anxiety disorders. Mid-course students had a higher frequency and intensity of substance use.

**Supplementary Information:**

The online version contains supplementary material available at 10.1186/s12909-022-03752-6.

## Background

Medical students have a high prevalence of mental disorders, especially anxiety disorders. In addition, they have a higher prevalence of stress, psychiatric, emotional disorders and suicidal ideation than the general population [1–5].

The main stressors are extensive content, lack of time to study, sleep deprivation, excessive self-pressure for good grades, lack of leisure time, insecurity in dealing with illness situations and facing life and death dilemmas prematurely [1]. The frustration is highlighted by the basic training cycle not directly related to medical practice, a large number of hospital activities, contact with patients with serious illnesses, and professional perspectives. Social aspects such as living far from home and the full-time course, also compromise leisure activities and social relationships [6, 7].

Furthermore, psychiatric disorders may be associated with reduced cognitive performance. Especially in anxiety disorders, impaired attention stands out as the main responsible for impaired cognitive performance, associated with symptoms that also affect thinking, perception and learning, such as restlessness, sweating, tachycardia, tremors, fatigue and sleep disorders [8]. Anxiety disorders are characterized by heightened feelings of fear and anxiety, leading to behavioral changes [9]. Anxiety can impair cognitive performance by physiological changes in the neural resources responsible for cognitive tasks, and by the overload of working memory resources with negative thoughts and ruminations [8].

In this sense, the use of alcohol and illicit drugs may result in or be induced by depressive and anxiety disorders, leading to cognitive impairment. Substance use disorders include cognitive, behavioral, and physiological symptoms associated with continued substance use. Substance-induced disorders consist of intoxication, withdrawal, and mental disorders directly induced by the substance, such as psychotic, depressive, and anxiety disorders [9].

By analyzing the connection between anxiety disorders and the use of alcohol and illicit substances on the academic performance of medical students, it will be possible to help in the development of proposals and measures to address these problems. The aim of the present study was to assess the presence of anxiety disorders and use of alcohol and illicit substances, and their influence on the academic performance of medical students at a public university.

## Method

### Study design and participants

This is a cross-sectional, non-experimental pilot study, with quantitative analyses, in which 67 medical students from the Federal University of Rondonópolis, Mato Grosso, Brazil were included.

Undergraduate students aged at least 18 years old, of both sexes, from the 1st to the 6th medical year, randomly selected, who signed and agreed with the Informed Consent Form, participated in the study. Students were invited through oral communication during classes, personally by the main researcher (PMF). A lottery was carried out for insertion in the study to minimize selection bias, aiming to include 12 students per class. Students were evaluated according to the year of the course, from 1 to 6; and according to the phases of the course, divided into basic cycle (1st and 2nd years), pre-clinical (3rd and 4th years) and clinical or internship cycle (5th and 6th years).

Data obtained from the instruments applied to assess anxiety disorders and use of alcohol and illicit substances in the medical students were related to the coefficient of academic performance.

The students were invited to participate in the study. They were informed about the research procedures and the confidentiality of the data collected. Student participation was voluntary and they did not receive any financial or other incentives. Students who did not understand any of the items in any of the assessment instruments were excluded from the study.

The study was approved by the Ethics Committees of the University of Campinas and the Federal University of Rondonópolis (CAAE 07,561,419.5.0000.5404).

### Application of instruments

The medical students were evaluated through the self-application of 2 non-invasive assessment instruments: 1) State-Trait Anxiety Inventory (STAI), to assess anxiety, and 2) ASSIST (Alcohol, Smoking and Substance Involvement Screening Test), to track the involvement with tobacco, alcohol and other substances. The instruments were completed by the students, under the supervision of the executing researcher (PMF), between June 2019 and March 2020.

### Applied instruments

#### State-Trait Anxiety Inventory (STAI)

STAI is a self-administered validated instrument developed to assess anxiety in normal adults. The application time is about 10 min and comprises 2 subscales, each consisting of 20 questions. The first scale assesses the state of anxiety, representing the individual's feeling when answering the questions. State anxiety reflects a personal characteristic in the response to stressful situations with anxiety and a tendency to perceive many situations as threatening. The second subscale assesses the trait anxiety, evaluating how the individual feels in general. The score was determined according to a Likert-type scale, from 1 to 4. In constructing the final score, the items referring to the factors “absence of state anxiety” and “absence of trait anxiety” were scored inversely. The final score was between 40 and 160 points. The cutoff point used was 40 points for each subscale, that is, individuals with 40 or more points in each subscale were considered to have significant clinical symptoms of anxiety [10]. Thus, higher scores obtained in the STAI-State instrument reflects a more intense state of anxiety [8].

#### ASSIST (Alcohol, Smoking and Substance Involvement Screening Test)

ASSIST is a validated instrument used for diagnostic screening of alcohol and other drug use, for detecting the frequency and intensity of substance use, and also for characterizing abuse or dependence, developed with the support of the World Health Organization (WHO). The application time is approximately 10 min, consisting of eight questions, with questions 1 to 7 addressing the use and problems related to various licit and illicit substances, and question 8 addressing the use of injecting drugs. Some additional drugs that are not on the list can be investigated in the category “other drugs”. The calculation of scores was performed by class of medications, adding the scores corresponding to questions 2 to 7, except tobacco, which does not apply to question 5. The need for non-intervention, brief intervention or intensive treatment was defined according to the score, for each class of medications [11]. For the statistical analysis, we chose to combine in two categories “non-intervention” and “intervention”, joining brief intervention with intensive treatment.

### Academic performance coefficient

This coefficient evaluates, throughout the course, the student's academic performance at the end of each academic period, and also, at the end of all semesters already attended, based on the students' accumulated grades. The value ranges from 0 (zero) to 10 (ten). The calculation was made from the weighted average of the sum of the grades of each subject multiplied by its value in credits, divided by the sum of the credits of the subjects, according to the formula:

$$\mathrm{CR}=\mathrm{ACADEMIC PERFORMANCE COEFFICIENT};\mathrm{CD}=\mathrm{SUBJECT CREDIT};\mathrm{N}=\mathrm{GRADE}$$

The calculation is done using the following formula:

$$\frac{\mathrm{CR}=\left({\mathrm{CD}}_{1} x {\mathrm{N}}_{1}\right) + \left({\mathrm{CD}}_{2} x {\mathrm{N}}_{2}\right)+\dots + \left({\mathrm{CD}}_{\mathrm{n}} x {\mathrm{N}}_{\mathrm{n}}\right)}{{\mathrm{CD}}_{1}+ {\mathrm{CD}}_{2} +\dots {\mathrm{CD}}_{\mathrm{n}}}$$

### Statistical analysis

The characteristics of the students were described according to the data obtained on anxiety and disorders caused by the use of alcohol and illicit substances and related to the academic performance coefficient. Tables of frequency were produced to assess the relationship between the categorical variables with the values of absolute frequency (n) and percentage (%). The relationship between the categorical and numerical variables was evaluated by obtaining descriptive measures (n, mean, standard deviation, minimum, median and maximum) of the numerical and in each category of the categorical, using for comparison the Mann–Whitney test.

The correlation between the numerical variables was verified by Spearman's coefficients, interpreted as positive or negative, following the scale: perfect (1.0), very strong (0.8 to 0.9), moderate (0.6 to 0.7), fair (0.3 to 0.5), poor (0.1 to 0.2) and absent (0) [12].

The significance level adopted for the study was 5%. The SAS System for Windows (Statistical Analysis System), version 9.4. SAS Institute Inc., 2002–2012, Cary, NC, USA, was used to perform the analysis.

## Results

Seventy-six medical students were recruited, of which 67 were included, obtaining a response rate of 88.15%. Specifically, 8 out of a total of 26 students from the 6th year (4 male and 4 female), 12 from 1st (total number = 37), 2nd (total number = 38), 4th (total number = 32) and 5th (total number = 33) years, 6 of each sex; and 11 students from 3rd (total number = 28) year, 5 male and 6 female.

Substance use by medical students occurred at the following rates: alcoholic beverages in 91.04% of them; tobacco and derivatives in 47.76% of them, marijuana in 35.82% of them, amphetamines or ecstasy in 17.91% of them and inhalants in 20.90% of the students. The other substances had a low frequency of previous use (data not shown).

Regarding the stage of the course, only the 4th year class had a higher prevalence of previous or current use of marijuana (*p* = 0.0184) and amphetamines or ecstasy (*p* = 0.001) than academics from other years. In relation to the other substances, there was no difference between the classes (data not shown).

As for the curricular stages, students in the Clinical cycle—Internship used less tobacco and its derivatives than the other classes (*p* = 0.0133). The classes in the pre-clinical cycle had a higher average consumption of amphetamines or ecstasy (*p* = 0.0006) and inhalants (*p* = 0.0256) than the other classes (data not shown).

Higher scores for trait anxiety (obtained by the STAI) among medical students were associated with a history of previous use of tobacco, marijuana, amphetamines or ecstasy and hypnotics and sedatives (obtained by the ASSIST). Trait anxiety and state score were related to the need for intervention for the use of amphetamines or ecstasy, as well as for trait anxiety score and need for intervention for use of hypnotics and sedatives (Table 1).

**Table 1:** Relation between State and Trait anxiety and prior licit and illicit substances use by medical students

Presence of substance use—ASSIST		STAI-State mean ± SD	*p*-value	STAI-Trait mean ± SD	*p*-value
Tobacco products	No (*n* = 35)	47.37 ± 11.25	0.5279	46.94 ± 11.97	0.2274
	Yes (*n* = 32)	48.41 ± 8.88		50.69 ± 12.61	
Tobacco products – Intervention	No (*n* = 56)	47.13 ± 10.47	0.1300	47.23 ± 12.10	0.0276
	Yes (*n* = 11)	51.64 ± 7.43		56.36 ± 11.02	
Alcohol	No (*n* = 6)	47.67 ± 13.76	0.8696	45.50 ± 16.67	0.4381
	Yes (*n* = 61)	47.89 ± 9.85		49.05 ± 11.96	
Alcohol – Intervention	No (*n* = 53)	47.47 ± 10.49	0.4075	48.60 ± 12.72	0.7998
	Yes (*n* = 14)	49.36 ± 8.82		49.21 ± 11.18	
Cannabis	No (*n* = 43)	47.40 ± 10.69	0.4544	46.37 ± 11.47	0.0466
	Yes (*n* = 24)	48.71 ± 9.19		52.96 ± 12.93	
Cannabis—Intervention	No (*n* = 65)	47.83 10.26	-	48.46 12.31	-
	Yes (*n* = 2)	49.00 5.66		57.50 13.44	
Cocaine and crack	No (*n* = 64)	47.78 ± 10.31	0.5262	48.58 ± 12.57	0.5165
	Yes (*n* = 3)	49.67 ± 5.69		52.00 ± 4.58	
Cocaine and crack—Intervention	No (*n* = 66)	47.91 ± 10.20	-	48.74 ± 12.43	-
	Yes (*n* = 1)	45.00 ± 0		48.00 ± 0	
Amphetamine-type stimulants	No (*n* = 55)	47.00 ± 10.25	0.1369	46.89 ± 11.69	0.0151
	Yes (*n* = 12)	51.83 ± 8.89		57.17 ± 12.13	
Amphetamine-type stimulants—Intervention	No (*n* = 63)	47.14 ± 9.73	0.0379	47.76 ± 11.84	0.0236
	Yes (*n* = 4)	59.25 ± 10.78		64.00 ± 10.80	
Inhalants	No (*n* = 53)	47.00 ± 10.63	0.1166	47.49 ± 12.17	0.1185
	Yes (*n* = 14)	51.14 ± 7.35		53.43 ± 12.22	
Inhalants—Intervention	No (*n* = 65)	47.40 ± 9.86	-	48.11 ± 11.98	-
	Yes (*n* = 2)	63.00 ± 8.49		69.00 ± 2.83	
Sedatives and sleeping pills	No (*n* = 57)	47.49 ± 10.51	0.3540	47.12 ± 12.28	0.0103
	Yes (*n* = 10)	50.00 ± 7.66		57.90 ± 8.17	
Sedatives and sleeping pills – Intervention	No (*n* = 58)	47.64 ± 10.48	0.5397	47.29 ± 12.24	0.0163
	Yes (*n* = 9)	49.33 ± 7.81		58.00 ± 8.66	
Hallucinogens	No (*n* = 61)	47.70 ± 10.16	0.7928	48.21 ± 12.06	0.3211
	Yes (*n* = 6)	49.50 ± 10.60		54.00 ± 14.99	
Hallucinogens—Intervention	No (*n* = 66)	47.86 ± 10.20	-	48.70 ± 12.42	-
	Yes (*n* = 1)	48.00 ± 0		51.00 ± 0	
Opioids	No (*n* = 65)	47.91 ± 10.28	-	48.71 ± 12.52	-
	Yes (*n* = 2)	46.50 ± 2.12		49.50 ± 2.12	
Opioids—Intervention	No (66)	47.86 ± 10.20	-	48.70 ± 12.42	-
	Yes (*n* = 1)	48.00 ± 0		51.00 ± 0	

Mann–Whitney Test

Trait anxiety score (obtained by STAI) showed a positive correlation with the use of tobacco and its derivatives (*ƿ* = 0.25821, poor), amphetamines or ecstasy (*ƿ* = 0.31782, fair) and hypnotics or sedatives (*ƿ* = 0.32528, fair) (obtained by ASSIST). State Anxiety score had a positive correlation with the alcohol use score (*ƿ* = 0.24754, poor) (Table 2).

**Table 2:** Correlations between state and trait anxiety of medical students with the use of tobacco and derivatives, alcoholic beverages and illicit substances

Presence of substance use—ASSIST	STAI score	
	State	Trait
	ƿ / *p*-value	
Tobacco products	0.20520	0.25821
	0.0957	0.0349
Alcohol	0.24754	0.21948
	0.0434	0.0743
Cannabis	0.17526	0.23816
	0.1560	0.0523
Cocaine and crack	0.00860	0.02689
	0.9449	0.8290
Amphetamine-type stimulants	0.23699	0.31782
	0.0535	0.0088
Inhalants	0.18708	0.21236
	0.1295	0.0845
Sedatives and sleeping pills	0.10919	0.32528
	0.3791	0.0072
Hallucinogens	0.02867	0.03503
	0.8179	0.7784
Opioids	0.00955	0.02757
	0.9388	0.8248

Spearman correlation coefficients: *ƿ* / *p*-value

### Academic performance

A lower achievement coefficient in the course was associated with previous use of tobacco and its derivatives, marijuana, amphetamines or ecstasy and hypnotics or sedatives. Additionally, worse academic performance was related with the need for intervention for the use of tobacco and its derivatives and hypnotics and sedatives (Table 3).

**Table 3:** Relationship between the use of tobacco, alcohol and illicit substances with the academic performance coefficient of medical students

Presence of substance use—ASSIST		ACADEMIC PERFORMANCE COEFFICIENT mean ± SD	*p*-value
Tobacco products	No (*n* = 35)	8.19 ± 0.47	0.0022
	Yes (*n* = 32)	7.67 ± 0.70	
Tobacco products—Intervention	No (*n* = 56)	8.06 ± 0.55	0.0011
	Yes (*n* = 11)	7.31 ± 0.73	
Alcohol	No (*n* = 6)	8.30 ± 0.47	0.1441
	Yes (*n* = 61)	7.91 ± 0.65	
Alcohol—Intervention	No (*n* = 53)	7.97 ± 0.61	0.5636
	Yes (*n* = 14)	7.80 ± 0.76	
Cannabis	No (*n* = 43)	8.11 ± 0.58	0.0020
	Yes (*n* = 24)	7.63 ± 0.64	
Cannabis—Intervention	No (*n* = 65)	7.99 ± 0.58	-
	Yes (*n* = 2)	6.32 ± 0.22	
Cocaine and crack	No (*n* = 64)	7.98 ± 0.61	0.0531
	Yes (*n* = 3)	7.07 ± 0.86	
Cocaine and crack—Intervention	No (*n* = 66)	7.96 ± 0.61	-
	Yes (*n* = 1)	6.16 ± 0	
Amphetamine-type stimulants	No (*n* = 55)	8.02 ± 0.60	0.0475
	Yes (*n* = 12)	7.57 ± 0.75	
Amphetamine-type stimulants—Intervention	No (*n* = 63)	7.98 ± 0.62	0.0626
	Yes (*n* = 4)	7.31 ± 0.81	
Inhalants	No (*n* = 53)	8.02 ± 0.60	0.0571
	Yes (*n* = 14)	7.62 ± 0.74	
Inhalants—Intervention	No (*n* = 65)	7.94 ± 0.65	-
	Yes (*n* = 2)	7.77 ± 0.42	
Sedatives and sleeping pills	No (*n* = 57)	8.00 ± 0.63	0.0138
	Yes (*n* = 10)	7.51 ± 0.59	
Sedatives and sleeping pills – Intervention	No (*n* = 58)	7.99 ± 0.63	0.0344
	Yes (*n* = 9)	7.55 ± 0.62	
Hallucinogens	No (*n* = 61)	7.96 ± 0.62	0.4509
	Yes (*n* = 6)	7.68 ± 0.85	
Hallucinogens—Intervention	No (*n* = 66)	7.94 ± 0.65	-
	Yes (*n* = 1)	7.87 ± 0	
Opioids	No (*n* = 65)	7.97 ± 0.61	-
	Yes (*n* = 2)	7.02 ± 1.21	
Opioids—Intervention	No (66)	7.94 ± 0.65	-
	Yes (*n* = 1)	7.87 ± 0	

Mann–Whitney Test

The coefficient of academic performance was negatively correlated with the score of use of tobacco and derivatives (*ƿ* = -0.42526, fair), alcoholic beverages (*ƿ* = -0.27385, poor), marijuana (*ƿ* = -0.32652, fair) and hypnotics and sedatives (*ƿ* = -0.30895, fair). Furthermore, this coefficient had a negative correlation with anxiety trait (*ƿ* = -0.35317, fair) (Table 4).

**Table 4:** Correlation between the academic performance of medical students with the use of substances licit and illicit and state and trait anxiety

VARIABLES	ACADEMIC PERFORMANCE COEFFICIENT
	ƿ / *p*-value
Presence of substance use—ASSIST	
Tobacco products	-0.42526
	0.0004
Alcohol	-0.27385
	0.0261
Cannabis	-0.32652
	0.0075
Cocaine and crack	-0.18264
	0.1422
Amphetamine-type stimulants	-0.19495
	0.1167
Inhalants	-0.21809
	0.0785
Sedatives and sleeping pills	-0.30895
	0.0116
Hallucinogens	-0.04884
	0.6970
Opioids	-0.17926
	0.1498
STAI	
State	-0.23301
	0.0597
Trait	-0.35317
	0.0036

(*Ƿ*) Spearman Correlation Coefficients / *P* value

## Discussion

The aim of this study was to identify factors that could negatively influence the academic performance of medical students, especially those related to the use of tobacco, alcohol and illicit substances, as well as behavioral changes, such as anxiety disorders.

We found worse academic performance correlated with more frequent use of tobacco products, alcoholic beverages, cannabis, sedatives and sleeping pills (hypnotics), in agreement with the literature, which suggests that the use of these substances is associated with anxiety and depression [13–16], which in turn are associated with cognitive impairment [8, 17]. Some of these substances, such as alcohol, cannabis and hypnotics/sedatives, are also known to be associated with cognitive impairment [18–22]. On the other hand, intense and prolonged use of tobacco can also cause cognitive impairment, however, in the short term occurs the opposite, a perception of cognitive improvement [23–26], characteristics not observed in this study.

In this sense, the worst school performance found among students with previous use of tobacco and derivatives, cannabis, amphetamine-type stimulants and hypnotics or sedatives can be explained by the association between psychiatric disorders and the use of these substances. In addition, the cognitive impairment caused by the acute and chronic effect of the use of these substances is highlighted.

The ASSIST instrument allows verifying that obtaining a higher score for a given substance indicates more intense and frequent use, pointing to the need for intervention by a professional in the area. Thus, students who consumed more intensely and frequently tobacco or its derivatives, hypnotics or sedatives had a higher prevalence of psychiatric disorders, which would clearly impair academic performance. Furthermore, the intense and frequent use of hypnotics or sedatives was associated with poorer academic performance in the short term, in agreement with the literature [18, 21].

In addition, poorer academic performance was correlated with a higher frequency of trait anxiety, probably explained by the impairment in the ability to maintain attention, commonly caused by anxiety. The trait anxiety refers to a relatively stable personal disposition to respond with anxiety to stressful situations and a tendency to perceive a greater number of situations as threatening [8]. Thus, the correlation between such personal characteristic could be recognized as a triggering factor for the worst school performance of these students.

The STAI instrument allowed us to identify that the more frequent and intense the use of tobacco and its derivatives, amphetamine-type stimulants and hypnotics or sedatives, the stronger the presence of anxiety. Thus, it is possible to infer causality in both directions, and demonstrate that the more intense use of these substances would be associated with the emergence and/or worsening of previous anxiety, as well as the presence of intense anxiety would lead to more intense use of these substances. The subjective feelings of tension seen in state anxiety can vary in intensity over time and be transient. Thus, the highest score obtained in the STAI-State instrument reflects a more intense state of anxiety [8].

We verified that the more intense and frequent consumption of alcoholic beverages was associated with a more accentuated state anxiety. The consumption of alcoholic beverages is widespread among university students [26, 27], and, as it is an easily accessible legal substance, there would be several periods of accentuation of alcohol consumption, probably triggered by transient episodes of emotional tension.

The pre-clinical phase of the course had a higher frequency and intensity of use of tobacco products, amphetamine-type stimulants and inhalants compared to the basic and clinical cycles. Students in the pre-clinical phase present uncertainties about the use of knowledge acquired in the basic cycle and, insecurity regarding the ability to manage the clinical cycle, which could predispose to substance use illegal as well as psychiatric disorders such as anxiety and depression. Still, in this period begins the clinical activity, more contact with patients and the medical activity itself, which brings doubts and uncertainties, such as the fear of not being able to satisfactorily act as a physician after completing the course [28, 29].

This study had some limitations. It was considered as a pilot study mainly due to the relatively small number of students included per class (ranging from 8 to 12 students), however, the total number of students belonging to each class is not high, ranging from 26 to 38, a limitation inherent to the characteristics of the university. In addition, students may have omitted or altered information from the ASSIST questionnaire for fear that the information would become public and/or that they would suffer some form of redress for the use of illegal substances. Finally, the study carried out in a single center could make it difficult to extrapolate the results to other courses of medicine.

As a strong point, this is a study that covered all years of the medical course, assessing substance use and anxiety disorder and the relationship of these factors with academic performance, a topic that has been little explored in the literature.

In conclusion, we were able to demonstrate that the frequent use of tobacco and its derivatives, alcoholic beverages, marijuana, hypnotics and sedatives was associated with worse academic performance in medical students. Allied to this finding, the state anxiety was correlated with the worst academic performance of these students. In addition, state and trait anxiety were associated with frequent use of tobacco and tobacco products, alcoholic beverages, marijuana, hypnotics and sedatives. Students in the pre-clinical phase of the medical course were more affected by both the state of anxiety and the tendency to use alcoholic beverages, tobacco and other drugs.

In view of these data, it is important to propose medical and psychological assessment and monitoring of medical students, in order to minimize the negative influences of emotional stress present in the period of medical training. However, further research is needed to confirm the findings of the study.

## Supplementary Information

**Additional file 1.**

## Data Availability

Authors confirm that all relevant data are included in the article.
