# Supplementary material for: Impact of the use of illicit and licit substances and anxiety disorders on the academic performance of medical students: a pilot study
Source: BMC Med Educ. 2022 Sep 19;22:684. doi: 10.1186/s12909-022-03752-6 (PMC9484241; doi:10.1186/s12909-022-03752-6)
Supplement: Supplementary file 1 — Additional file 1. [file 12909_2022_3752_MOESM1_ESM.docx]

**IMPACT OF THE USE OF ILLICIT AND LICIT SUBSTANCES AND ANXIETY DISORDERS ON THE ACADEMIC PERFORMANCE OF MEDICAL STUDENTS: A PILOT STUDY.**

**SUPPLEMENTARY DATA**

Table 1 - Current or previous use of alcoholic beverages, tobacco and its derivatives, illicit substances in medical students.

| Substance Use | Frequency N (%) |
| --- | --- |
| Tobacco products | 32 (47.76) |
| Alcohol | 61 (91.04) |
| Cannabis | 24 (35.82) |
| Cocaine and crack | 3 (4.48) |
| Amphetamine-type stimulants | 12 (17.91) |
| Inhalants | 14 (20.90) |
| Sedatives and sleeping pills | 10 (14.93) |
| Hallucinogens | 6 (8.96) |
| Opioids | 2 (2.99) |

Data obtained by ASSIST instrument

Table 2. Comparison of prior substance use among the 1st to 6th- year classes of medical students.

| Presence of substance use | Course year N = 67(%) | | | | | | p-value |
| --- | --- | --- | --- | --- | --- | --- | --- |
|  | 1 (n=12) | 2 (n=12) | 3 (n=11) | 4 (n=12) | 5 (n=12) | 6 (n=8) |  |
| Tobacco products | 5 (15.62) | 6 (18.75) | 7 (21.88) | 6 (18.75) | 6 (18.75) | 2 (6.25) | 0.6964 |
| Alcohol | 9 (14.75) | 12 (19.67) | 11 (18.03) | 11 (18.03) | 12 (19.67) | 6 (9.84) | 0.0651 |
| Cannabis | 2 (8.33) | 5 (20.83) | 8 (33.33) | 4 (16.67) | 5 (20.83) | 0 (0) | 0.0184* |
| Cocaine and crack | 0 (0) | 1 (33.33) | 1 (33.33) | 1 (33.33) | 0 (0) | 0 (0) | 0.8557 |
| Amphetamine-type stimulants | 0 (0) | 1 (8.33) | 7 (58.33) | 1 (8.33) | 1 (8.33) | 2 (16.67) | 0.0010* |
| Inhalants | 0 (0) | 3 (21.43) | 5 (35.71) | 2 (14.29) | 2 (14.29) | 2 (14.29) | 0.1610 |
| Sedatives and sleeping pills | 2 (20.00) | 1 (10.00) | 3 (30.00) | 3 (30.00) | 0 (0) | 1 (10.00) | 0.4085 |
| Hallucinogens | 0 (0) | 1 (16.67) | 3 (50.00) | 0 (0) | 1 (16.67) | 1 (16.67) | 0.2127 |
| Opioids | 0 (0) | 1 (50.00) | 1 (50.00) | 0 (0) | 0 (0) | 0 (0) | - |

Data obtained by ASSIST instrument. Kruskal-Wallis tests and Multiple comparisons made by Dunn's test. *4th year class higher than other years

Table 3. Comparison of prior substance use among medical students from basic, clinical and internship cycles.

| Presence of substance use – ASSIST | Course year (=67) (mean ±SD) | | | p-value |
| --- | --- | --- | --- | --- |
|  | Basic cycle (n=24) | Preclinical cycle (n=23) | Clinical cycle – Internship (n=20) |  |
| Tobacco products | 2.5 ± 4.57 | 4.22 ± 8.82 | 0.0 ± 0.0 | 0.0133^A^ |
| Alcohol | 6.63 ± 6.90 | 6.13 ± 4.91 | 4.75 ± 6.09 | 0.2764 |
| Cannabis | 0.54 ± 1.41 | 1.74 ± 6.24 | 0.0 ± 0.0 | 0.0996 |
| Cocaine and crack | 0.13 ± 0.61 | 0.26 ± 1.25 | 0.0 ± 0.0 | 0.6480 |
| Amphetamine-type stimulants | 0.0 ± 0.0 | 1.30 ± 2.36 | 0.0 ± 0.0 | 0.0006^B^ |
| Inhalants | 0.08 ± 0.41 | 0.91 ± 2.11 | 0.0 ± 0.0 | 0.0256^C^ |
| Sedatives and sleeping pills | 1.25 ± 4.40 | 1.78 ± 3.77 | 0.75 ± 3.35 | 0.1699 |
| Hallucinogens | 0.17 ± 0.82 | 0.0 ± 0.0 | 0.0 ± 0.0 | 0.4083 |
| Opioids | 0.17 ± 0.82 | 0.13 ± 0.63 | 0.0 ± 0.0 | 0.6492 |

Kruskal-Wallis test and Multiple comparisons made by Dunn's test. ^A^ASSIST- Tobacco products Score: Basic cycle lower than other classes. ^B^ASSIST- Amphetamine-type stimulants Score: Preclinical cycle: higher average than other classes. ^C^ASSIST-Inhalants Score: Average Preclinical cycle higher than other classes.
